# Differentiating Sinonasal Tumor Entities with Fluorescein-Enhanced Confocal Laser Endomicroscopy: A Step Forward in Precision Diagnostics

**DOI:** 10.3390/cancers16244245

**Published:** 2024-12-20

**Authors:** Nina Wenda, Sebastian Wagner, Kai Fruth, Annette Fisseler-Eckhoff, Jan Gosepath

**Affiliations:** 1 Department of Otolaryngology, Head and Neck Surgery, Helios HSK Wiesbaden, 65199 Wiesbaden, Germany; kai.fruth@web.de (K.F.); jan.gosepath@helios-gesundheit.de (J.G.); 2 Department of Pathology, Helios HSK Wiesbaden, 65199 Wiesbaden, Germany; sebastian.wagner@helios-gesundheit.de (S.W.); annette.fisseler-eckhoff@helios-gesundheit.de (A.F.-E.)

**Keywords:** confocal laser endomicroscopy, optical biopsy, sinonasal tumors

## Abstract

**Abstract: Background/Objectives**: Sinonasal malignancies are rare and highly diverse cancers that pose significant diagnostic challenges due to their variable histological features and complex anatomical locations. Accurate diagnosis is critical for guiding treatment, yet conventional methods often require multiple biopsies. This study aimed to evaluate the potential of confocal laser endomicroscopy (CLE) for real-time imaging of sinonasal tumors to characterize specific features of different entities and improve diagnostic precision. **Methods**: Ten patients with various sinonasal malignancies, including squamous cell carcinoma, adenocarcinoma, sinonasal undifferentiated carcinoma, olfactory neuroblastoma, sinonasal mucosal melanoma, and endonasal lymphoma, were examined using CLE during diagnostic endoscopy. CLE images were compared descriptively with histopathological cross-sections to identify unique imaging patterns for each tumor type. **Results**: CLE was feasible across all cases, with high-quality images obtained despite anatomical challenges in some cases. Characteristic features, such as vascular clusters in undifferentiated carcinoma, mucin-filled bubbles in adenocarcinoma, and small round cells in neuroblastoma, were identified and corresponded well with histopathological findings. CLE also helped guide biopsies by revealing areas with diagnostic relevance. **Conclusions**: CLE demonstrates promise as an adjunct diagnostic tool in sinonasal malignancies, offering real-time imaging that correlates with histopathological findings and aids in targeted biopsies. While this study provides preliminary insights into the utility of CLE, further research with larger cohorts and statistical validation is necessary to establish its diagnostic reliability and broader clinical application.

## Simple Summary

Sinonasal tumors are rare and diverse cancers that are challenging to diagnose and treat. These tumors require precise identification because treatment decisions depend heavily on their specific type. In this study, we explored the use of confocal laser endomicroscopy (CLE), a technique that allows real-time imaging of tissues during surgery or diagnostic procedures, to better understand how it could help in diagnosing these tumors. By comparing CLE images with traditional tissue analysis, we identified unique features for different tumor types, including squamous cell carcinoma, adenocarcinoma, and sinonasal malignant melanoma. CLE also helped guide biopsies, potentially reducing the need for repeat procedures. While further studies are needed to confirm our findings in larger groups, this research provides a foundation for using CLE to improve the accuracy of sinonasal cancer diagnosis and treatment, benefiting both patients and the research community.

## 1. Introduction

Sinonasal malignancies represent a rare and diverse group of tumors arising within the nasal cavity and paranasal sinuses [1]. These malignancies encompass a wide spectrum of histopathological entities and pose significant diagnostic and therapeutic challenges [2]. Treatment strategies primarily depend on the histopathological type, which dictates the prognosis and therapeutic approach. Surgical resection is generally the cornerstone of treatment for resectable tumors, with adjuvant therapies tailored according to the specific tumor type and staging [3]. Accurate preoperative diagnosis and intraoperative evaluation are crucial in guiding treatment decisions.

Confocal laser endomicroscopy (CLE) has emerged as a valuable tool in various specialties, allowing for real-time, in vivo visualization of cellular structures, often referred to as “optical biopsies” [4]. Initially developed within gastroenterology, CLE is extensively used in both diagnostic and therapeutic pathways for malignant and inflammatory diseases of the gastrointestinal tract [5,6]. Its success in gastroenterology has led to expanding CLE’s application to other specialties, including pulmonology [7,8], urology [9], gynecology [10], general surgery [11], and neurosurgery [12]. In recent years, its application in the head and neck region has been explored, with promising results in delineating malignant from healthy tissue, aiding in surgical margin assessment, and potentially reducing the need for additional invasive biopsies [13,14,15]. Despite these advances, CLE’s application within sinonasal malignancies, a domain characterized by its tumor diversity and anatomical complexity, remains relatively unexplored.

Given the rarity and histological variability in sinonasal tumors, CLE may offer benefits in supporting diagnostic and therapeutic decision-making. Unlike previous studies, which predominantly focus on CLE’s utility in distinguishing malignant from healthy tissue, our study aims to assess whether CLE can also aid in differentiating among various sinonasal tumor entities in vivo.

If successfully integrated into clinical workflows, CLE could offer significant benefits for diagnosing sinonasal malignancies by providing real-time, in vivo imaging. This technology can help identify tumor-specific features such as vascularization and cellular morphology while also assessing surrounding mucosa for infiltration, which is essential for surgical planning. Additionally, CLE can guide biopsies by avoiding necrotic areas and targeting cell-rich regions, ensuring adequate material is obtained for accurate diagnosis. These capabilities may reduce the need for repeated procedures and enable faster, more precise treatment decisions.

In this study, we investigated the application of CLE in a cohort of patients with diverse sinonasal malignancies, including squamous cell carcinoma (SCC), adenocarcinoma AC), sinonasal malignant melanoma (SNMM), sinonasal undifferentiated carcinoma (SNUC), olfactory neuroblastoma (ONB), and sinonasal lymphoma (SL). Our primary aim was to evaluate whether CLE could support the diagnostic process in these rare tumors by identifying distinguishing imaging characteristics across these specific tumor types. Understanding whether distinct CLE imaging patterns correlate with individual tumor histologies could lead to more precise, real-time diagnostic insights and potentially influence the surgical strategy. We believe this approach could represent a novel step toward refining the optical biopsy capabilities of CLE for sinonasal malignancies, enhancing diagnostic precision.

## 2. Materials and Methods

This study aimed to investigate the application of CLE in the diagnostic assessment and endoscopic resection of diverse sinonasal malignancies. Ten patients with different histological types of endonasal tumors—including SCC, AC, SNMM, SNUC, ONB, and SL—were enrolled from a tertiary medical center in Germany. This study was conducted in accordance with the principles outlined in the Declaration of Helsinki, and approval was obtained from the institutional review board (IRB). Written informed consent was obtained from all participants prior to inclusion in this study.

Patient demographics and tumor characteristics for this study included a cohort of ten individuals diagnosed with various types of sinonasal malignancies. Among the three patients with SCC, there were two male patients aged 68 and 75 years and one female patient aged 56 years. Two patients presented with SNMM: one female aged 58 years and one male aged 84 years. The AC cohort included one female patient aged 83 years and one male patient aged 57 years. The SNUC case involved a 51-year-old male, while the ONB case was a 55-year-old male. Finally, the single case of SL was identified in a 49-year-old female. This demographic diversity enabled us to investigate CLE imaging across a range of sinonasal malignancy types and patient profiles.

In CLE, a low-power laser targets a specific point within a microscopic field of view to capture high-resolution images of in vivo tissue. Focused laser light of a defined wavelength is passed through a confocal aperture, allowing the microscope to capture and reconstruct images in two dimensions. In this setup, the laser scanning unit and light source are external to the body, with the laser beam transported through flexible confocal miniprobes containing a bundle of over 10,000 optical fibers. These miniprobes, which have a diameter of 1.6 mm, sequentially scan the laser beam, allowing for two-dimensional image reconstruction and generation of grayscale images.

We used the Cellvizio system by Mauna Kea Technologies (Paris, France). In this setup, the laser scanning unit and light source are external to the body, with the laser beam transported through flexible confocal miniprobes containing a bundle of over 10,000 optical fibers. These miniprobes, which have a diameter of 1.6 mm, sequentially scan the laser beam, allowing for two-dimensional image reconstruction.

The generated CLE images are displayed in a horizontal plane, which is perpendicular to the conventional perspective of histopathological cross-sections. This difference in orientation is important for accurately comparing and analyzing the CLE images alongside histopathological images, as it requires a reorientation of perspective to correlate structural details between the two imaging modalities.

CLE data are collected at a rate of 12 frames per second, enabling video-quality imaging and real-time visualization down to the level of individual erythrocytes within the blood vessels. The field of view is 240 µm^2^, with a confocal depth range of 55–65 µm, with each image acquired at a fixed depth within this range. This setup provided a high lateral resolution of 1 µm, an axial resolution of 5 µm, and a magnification of up to 1000-fold, allowing for detailed examination of cellular and vascular structures within the nasal cavity and the paranasal sinuses.

Intravenously administered Fluorescein was used as the contrast agent due to its affordability, non-mutagenic properties, and established safety profile.

The workflow for CLE examination started with conventional endoscopy to visualize the tumor and assess its location and general characteristics. Once the tumor was identified, an intravenous injection of 2.5 mL of a 10% fluorescein solution was administered. After a waiting period of approximately 30 s to allow fluorescein to circulate, CLE was used to examine the tumor and the surrounding mucosa. This allowed for the identification of promising biopsy sites based on features such as cellular morphology, vascular patterns, and tissue architecture. Biopsies were then performed, targeting these selected areas to maximize diagnostic yield. This approach ensured that tissue samples were taken from regions most likely to provide diagnostically valuable information.

All procedures were performed under general anesthesia to ensure patient comfort and optimal procedural conditions. The use of CLE added up to 10 min to the overall procedure time, depending on the localization and accessibility of the tumor within the sinonasal cavity.

**Figure 1:**
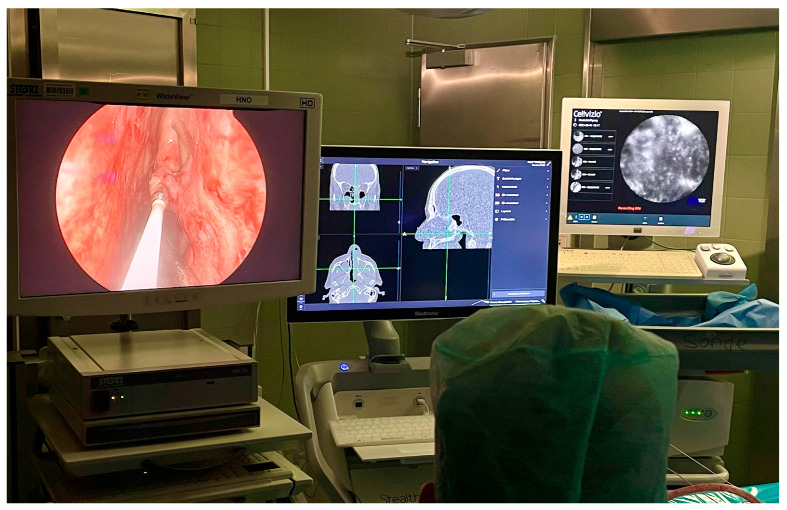
Intraoperative setup from the surgeon’s perspective, arranged from left to right. The endoscopic image shows the laser probe positioned on the inferior turbinate, electromagnetic navigation system (Stealth Station^®^, Medtronic, Jacksonville, FL, USA), and confocal laser endomicroscope (Cellvizio^®^, Mauna Kea Technologies, Paris, France).

Figure 1 displays the diagnostic setup during endonasal endoscopy.

During endoscopy, approximately 5 min of CLE video footage was captured for each patient, with imaging performed from multiple angles around the tumor to ensure comprehensive coverage. From this footage, several hundred images were extracted. Due to the presence of artifacts and variations in image quality, we selected 20 high-quality images per patient for further analysis. These selected images were then presented to two independent pathologists, alongside the final histopathological results, to enable a descriptive comparison and analysis of the CLE images with the histological images obtained through H&E staining.

**Figure 2:**
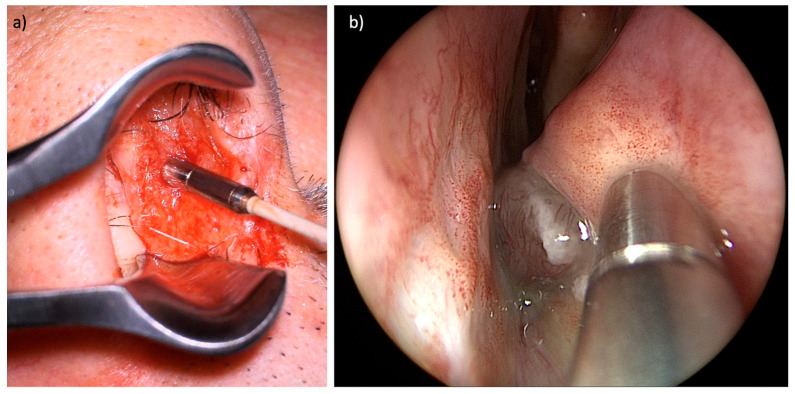
Positioning of the laser probe. (**a**) Examination of an SCC of the right nasal septum. (**b**) Examination of an SNMM of the left inferior turbinate.

Figure 2 shows an example of the placement of the laser probe in an SCC and an SNMM.

## 3. Results

The use of CLE was reliably achievable during diagnostic endoscopy with biopsy in all ten patients with various types of sinonasal tumors. CLE imaging commenced approximately 30 s after intravenous administration of fluorescein, and no unexpected side effects from the contrast agent were observed. The CLE examination added approximately 10 min to each procedure.

Image quality varied depending on factors such as tumor location, tissue vascularization, and tissue integrity, yet high-quality images were successfully obtained in all examined regions. The placement and positioning of the laser probe were manageable for imaging the nasal septum, the nasal cavity floor, and the tumors themselves. Additionally, CLE examination was achievable in more complex and constrained regions, including the paranasal sinuses—the ethmoid, maxillary, and sphenoid sinuses—as well as along the skull base, including the frontal recess. The use of rigid instruments facilitated precise probe placement in tight areas, such as the lateral nasal wall and regions beneath the remnants of the inferior turbinate.

As previously explained, it is important to remember that the perspectives of CLE images and histopathological cross-sections are not directly aligned; rather, they are perpendicular to one another. We remind the reader of this distinction here to clarify the basis for the following image analysis comparisons.

### 3.1. Healthy Mucosa

**Figure 3:**
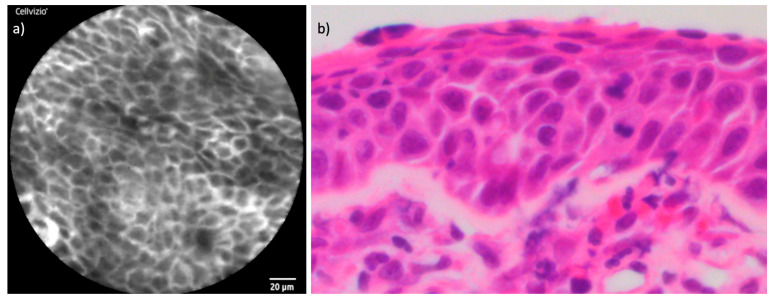
Juxtaposition of CLE and histopathological cross-section in healthy mucosa: (**a**) CLE image of endonasal mucosa of the inferior turbinate; (**b**) regular endonasal squamous epithelium with hematoxylin and eosin (H&E) staining.

**Figure 4:**
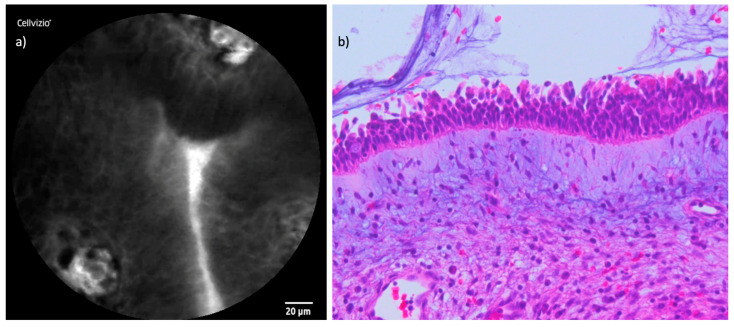
Comparison of CLE and histopathological cross-section respiratory epithelium: (**a**) CLE image of the endonasal respiratory epithelium of the nasal septum with cross-sections of capillaries. (**b**) Corresponding respiratory epithelium, also with cross-sections of capillaries in hematoxylin and eosin (H&E).

The examination of the healthy mucosa surrounding the tumors revealed uniform and characteristic features. In terms of cellular configuration, the healthy mucosa exhibited a regular, oval to polygonal architecture with clearly defined borders. Capillaries appeared small, with a round to oval shape, and were individually distinguishable without any evidence of perivascular fluorescein leakage. The uptake of fluorescein was homogeneously distributed throughout the healthy tissue, providing a uniform contrast that allowed clear visualization of cellular and vascular structures. Figure 3 and Figure 4 demonstrate the CLE findings of healthy mucosa and respiratory epithelium compared to corresponding histopathological cross-sections.

### 3.2. Squamous Cell Carcinoma

The application of CLE in SCC proved feasible and informative. Among the SCC cases, we examined one located in the nasal vestibule and two within the paranasal sinuses. In the nasal vestibule, the positioning of the CLE probe was straightforward, enabling the acquisition of high-quality images with minimal difficulty. In the paranasal sinuses, while the CLE examination was similarly achievable, the positioning of the probe was more challenging due to the complex anatomy. However, using rigid instruments facilitated precise probe placement in these regions, allowing for successful imaging.

Upon examining the SCC cases, we consistently observed distinctive structural changes within the tumor tissue. The cellular shape appeared deformed, with borders ranging from blurry to nearly indistinct. Capillaries showed irregular shapes and frequently formed vascular clusters, a feature not typically observed in healthy tissue. Perivascular fluorescein leakage was also evident, contributing to an inhomogeneous distribution pattern. In these areas, fluorescein uptake displayed brighter regions around the leakage sites, contrasting with darker regions where fluorescein uptake was reduced.

The juxtaposition of CLE images with histopathological cross-sections revealed a number of parallels, facilitating a robust comparison between the imaging modalities.

**Figure 5:**
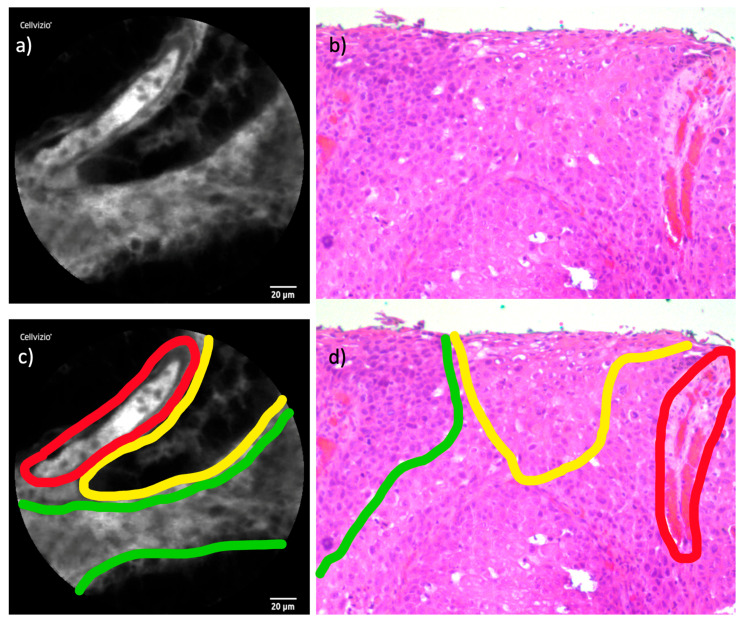
Comparison of CLE and histopathological cross-section in SCC: (**a**) CLE image of an endonasal SCC with blurry cell borders, irregular cell configuration, and inhomogeneous distribution of fluorescein. (**b**) Corresponding histopathological cross-section H&E staining. (**c**,**d**) Highlighted parallels in both modalities: **red:** cross-section of capillary, **yellow:** area of tumor necrosis without contrast enhancement, **green:** tumor cells with contrast enhancement.

Figure 5 shows a similar comparison of the two modalities in a SCC patient.

### 3.3. Sinonasal Adenocarcinoma

**Figure 6:**
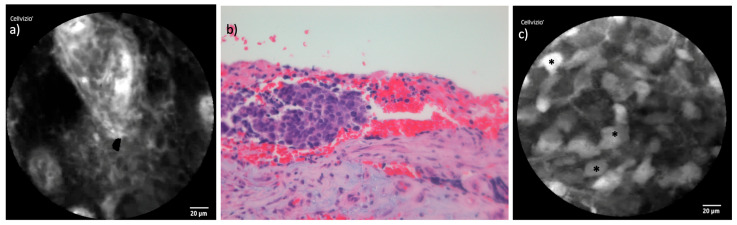
Comparison of CLE and histopathological cross-section in AC: (**a**) CLE image of one of the examined endonasal AC with a highly contrasted cluster of tumor cells with surrounding stromal desmoplasia with irregular cellular architecture and fluorescein leakage. (**b**) Corresponding histopathological cross-section demonstrating the clustered tumor cells as well. (**c**) CLE image from another area of the tumor with mucin-filled cells (*).

CLE was readily applicable in both cases of intestinal-type adenocarcinoma (ITAC) examined, both of which involved patients with a history of woodworking. Compared to other carcinoma in the cohort, AC exhibited relatively low vascularization, which reduced image artifacts and facilitated quicker acquisition of high-quality images, as less blood on the tumor surface allowed for clearer visualization. A unique finding in this tumor type was the presence of clusters of mucin-filled cells, likely reflecting the glandular structures characteristic of ITAC. This feature distinguished AC from other malignancies in this study and may represent a specific pattern recognizable in CLE imaging. Figure 6 displays the juxtaposition of CLE and histopathology in one of the examined AC patients.

### 3.4. Sinonasal Undifferentiated Carcinoma

**Figure 7:**
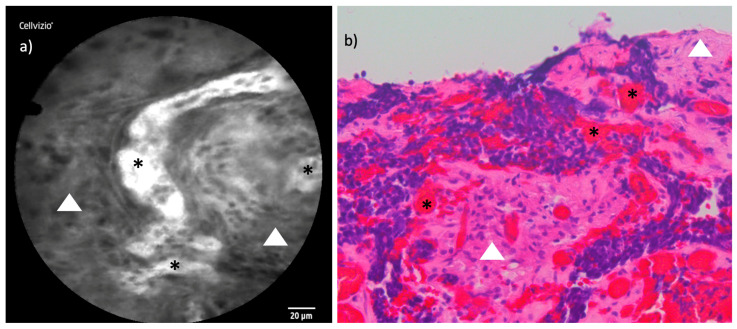
Comparison of CLE and histopathological cross-section in SNUC: (**a**) CLE image of an endonasal SNUC with an increased number of irregularly shaped, cluster-forming capillaries (*) in contrast to less contrasted stromal desmoplasia (white triangles). (**b**) Corresponding histopathological cross-section H&E staining.

The examination of the SNUC proved to be the most challenging among all tumor types in this study. This was due, in part, to the tumor’s anatomical location along the skull base and within the frontal sinus, areas that were particularly difficult to access with the laser probe. Additionally, the tumor had extensive vascularization, a common feature of this tumor type, which complicated the visualization of individual tumor cells. Unlike other tumors in the cohort, the distinguishing characteristic in this SNUC case was not the shape or arrangement of the tumor cells but rather the vascular patterns. We observed extensive hyperplastic capillaries and the formation of vascular clusters, features that were distinct and identifiable through CLE imaging despite the overall imaging challenges. Figure 7 demonstrates an exemplary CLE image compared to a respective histopathological cross-section of the SNUC case.

### 3.5. Olfactory Neuroblastoma

**Figure 8:**
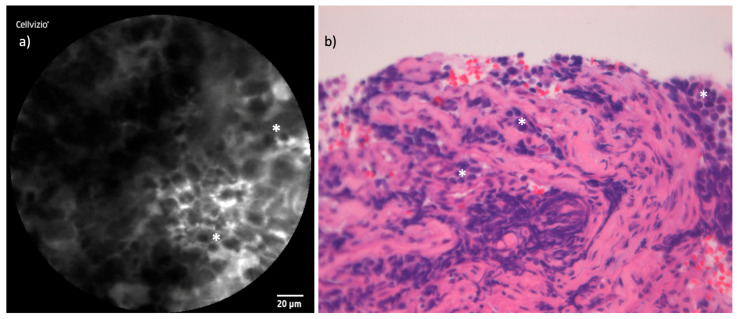
Comparison of CLE and histopathological cross-section in ONB: (**a**) CLE image of ONB with tumor clusters of small spheric cells (white *). (**b**) Corresponding histopathological cross-section H&E staining showing the typical small round blue appearance of the tumor cells (white *).

The examination of the ONB, which characteristically arises from the skull base, presented additional challenges in accessing the tumor site with the laser probe compared to tumors located in the nasal cavity or ethmoid sinus. However, by utilizing angled rigid instruments, such as a 45°-angled Blakesley forceps, we were able to stabilize the probe and achieve adequate image quality. ONB is classified as a small round cell tumor, and this characteristic cellular shape was consistently visible in the CLE images. These observations provided a reliable basis for distinguishing ONB from other tumor types in the cohort. Figure 8 compares the two modalities in two exemplary images of the ONB case.

### 3.6. Sinonasal Malignant Melanoma

**Figure 9:**
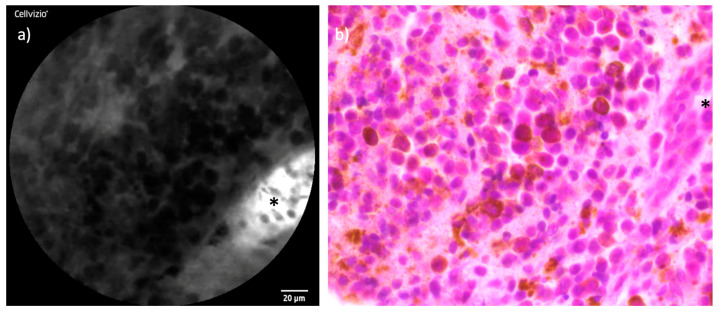
Comparison of CLE and histopathological cross-section in SNMM: (**a**) CLE image of one of the included SNMM with small round cells and a cross-section of a capillary (*). (**b**) Corresponding histopathological cross-section H&E staining showing the highly similar round cellular shape. The brown melanin pigment is not detected in the CLE image.

In the endoscopic inspection, both cases of SNMM displayed light pigmentation, which initially suggested SNMM as a potential diagnosis. CLE examination of the tumors revealed small, spherical cells, a finding that corresponded well with the histological images. However, while the histopathological analysis identified melanin pigments within the tumor tissue, these pigments were not detectable using CLE. Figure 9 shows the comparison of exemplary CLE and histopathological images of SNMM.

### 3.7. Sinonasal Lymphoma

**Figure 10:**
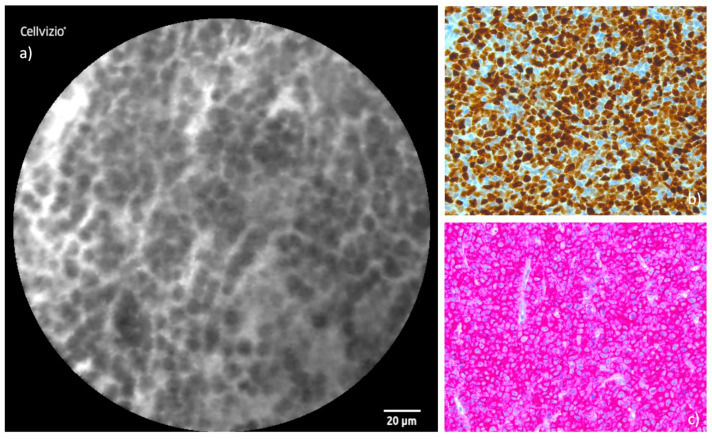
Comparison of CLE and histopathological cross-section in SL: (**a**) CLE image of SL with characteristically uniform clusters of spherical cells. (**b**) Corresponding histopathological cross-section with Ki67 immunostaining. (**c**) Corresponding histopathological cross-section with CD-20 immunostaining displaying the conformity of the modalities.

The examination of the SL presented challenges due to the tumor’s soft and easily disintegrating consistency, which made probe stabilization more difficult during CLE imaging. Despite this, the cellular shape observed in CLE images was uniformly spherical, correlating well with the histological findings. Notably, the uniform accumulation of these cells was a unique feature among the cohort, distinguishing the lymphoma from other tumor types examined in this study. Figure 10 displays the juxtaposition of CLE and histological images with immunostaining.

## 4. Discussion

### 4.1. Technology and Performance of CLE

CLE itself can be considered a safe procedure [16]. In our study, no adverse effects were observed following the administration of fluorescein, which has an established safety profile and has been widely used in medical practice for decades [17].

Achieving good image quality with CLE can be challenging, particularly in the initial stages of using the technology. The flexible properties of the probe present both advantages and disadvantages. While flexibility allows for adaptability in different anatomical regions, it requires precise handling to achieve optimal imaging. Reaching areas such as the maxillary sinus, frontal sinus, or skull base necessitates adjusting the angle of the probe in relation to the endoscope used to visualize the tumor. Angled instruments, such as a 45° Blakesley forceps or a Heuwieser antrum forceps, can aid in properly positioning the probe. These tools can help to achieve the correct contact angle and maintain appropriate contact pressure, both of which are critical for obtaining high-quality images.

CLE’s small field of view and limited imaging depth represent notable limitations, particularly in anatomically complex regions such as the sinonasal tract. These factors may restrict the ability to obtain a complete overview of larger or deeply situated lesions. These factors may require additional time and effort to ensure a comprehensive examination of the tumors and surrounding tissue, emphasizing the importance of precise probe positioning.

Most CLE studies, including our own, were conducted in tertiary care centers, and the procedures were frequently performed by expert endoscopists and surgeons. While this ensures optimal handling and accurate interpretation of the results, it limits the generalizability of data regarding procedural time and ease of handling. The learning curve for less experienced practitioners or those in non-tertiary settings may differ, potentially impacting the broader applicability of CLE in routine clinical practice.

The integration of CLE into pathology workflows also presents challenges due to the technical demands on both surgeons and pathologists. Head and neck pathology is a highly specialized field, and the use of CLE requires a learning curve for both disciplines. In our opinion, the best strategy to ensure the effective implementation of this technology is a collaborative approach, with surgeons and pathologists learning together. Bringing the pathologist into the operating room during CLE procedures can facilitate real-time correlation between CLE findings and histopathology, enhancing diagnostic accuracy. This collaborative learning process not only improves individual proficiency but also strengthens the integration of CLE into routine clinical practice. However, this requirement for additional expertise may limit the operational viability of CLE in settings with less specialized staff or resources.

Our findings highlight CLE’s potential to complement traditional diagnostic approaches by improving the diagnostic accuracy and efficiency of biopsies. CLE offers specific benefits during an endoscopic examination, such as providing an initial indication of the tumor entity by visualizing tumor-specific features like vascularization and cellular structure. Additionally, CLE allows for the evaluation of surrounding mucosa to determine whether it is infiltrated, which is crucial for surgical planning. In hematopoietic tumors, CLE can guide the extent of biopsies to ensure adequate material for immunostaining. Moreover, CLE’s ability to identify necrotic areas and direct biopsies to cell-rich regions enhances its diagnostic utility. These benefits underscore CLE’s potential impact in streamlining the diagnostic workflow for sinonasal malignancies. 

### 4.2. Use of CLE in Sinonasal Malignancies

Sinonasal malignancies represent a rare and highly diverse group of tumors, posing significant challenges for diagnosis and treatment [1]. The rarity of these malignancies, combined with their histological variability, means that solid, universally accepted treatment guidelines are lacking and are unlikely to be established in the foreseeable future [18]. As our understanding of these tumors evolves, new entities, such as DEK::AFF2 carcinoma, are being identified, further expanding the spectrum of sinonasal malignancies [19]. Additionally, parallels can be drawn to oropharyngeal carcinoma, as the role of human papillomavirus (HPV) in the pathogenesis of sinonasal malignancies is becoming increasingly recognized [20]. These advancements underscore the need for innovative diagnostic tools, such as CLE, to support individualized and precise management of these complex cases.

While CLE offers considerable advantages in real-time imaging and precise diagnostic capabilities, there are inherent limitations to the technology that must be acknowledged. One notable limitation is the increased procedural cost and time associated with its use. In our study, the performance of CLE added approximately 10 min to the diagnostic procedure. In comparison, a study on CLE in Barrett’s esophagus (BE) reported a procedural time of 18 min. However, the time required to perform CLE-targeted biopsies was 7.5 min shorter than that required for random biopsies, demonstrating the efficiency of targeted sampling [21]. Another study likely demonstrated that CLE improves diagnostic accuracy and reduces the number of biopsies required during surveillance for BE-associated dysplasia [22]. Similarly, using CLE to examine the periphery and center of tumors in our cohort revealed that tumor cells were not visible at every site within the tumor. This underscores the value of the additional time investment, as it facilitates more precise and informative biopsies, potentially avoiding the need for repeated sampling due to non-significant biopsy results.

#### 4.2.1. Squamous Cell Carcinoma

The use of CLE in the head and neck region has been most extensively studied in SCC [23]. Numerous studies have identified consistent malignancy criteria for SCC using CLE, including tissue homogeneity, cell size, cell borders, clustering, capillary loops, and fluorescein leakage [24,25,26].

A meta-analysis encompassing six studies with 213 patients and a total of 361 lesions examined with CLE in oral squamous cell carcinoma (OSCC) demonstrated the high diagnostic accuracy of this technique. The pooled outcomes showed excellent sensitivity at 95% (95% CI, 92–97%; I^2^ = 77.5%) and specificity at 93% (95% CI, 90–95%; I^2^ = 68.6%) [27].

The results of our study align with these findings, as we were able to visualize the malignancy criteria in all three cases of sinonasal SCC in our cohort. Furthermore, these properties were also identifiable in the comparison with histopathological cross-sections, reinforcing the diagnostic correlation between CLE and traditional histopathology. 

#### 4.2.2. Adenocarcinoma

Adenocarcinomas are the second most common malignancy of the sinonasal region, representing 10% to 20% of all sinonasal malignancies. These tumors can be classified into intestinal type (ITAC), nonintestinal type (non-ITAC), and salivary type [28]. The use of CLE in AC has not been extensively studied. In our cohort, we examined two cases of ITAC, both in woodworkers, a population with known occupational risk factors for this malignancy.

Compared to other tumors in this study, the AC cases displayed lower vascularization, resulting in fewer imaging artifacts and faster acquisition of high-quality images. A notable feature observed in these cases was the presence of clusters of mucin-filled bubble-like cells due to the glandular activity characteristic of this entity. This unique feature allowed for clear differentiation from other malignancies in the cohort.

#### 4.2.3. Sinonasal Undifferentiated Carcinoma

Sinonasal carcinomas are rare malignancies of the head and neck region, with an annual incidence of approximately 0.5 to 1 case per 100,000 population. Recent advances in molecular genetics have significantly enhanced our understanding of these neoplasms [29]. These advances have led to substantial revisions in their classification, as reflected in the fifth edition of the World Health Organization Classification of Head and Neck Tumors [30].

In our study, SNUC was the most challenging tumor to examine using CLE, primarily due to its anatomical location along the skull base and within the frontal sinus, which complicated probe positioning. Additionally, the pronounced vascularization characteristic of this tumor type introduced artifacts that made visualization of tumor cells difficult. Despite these challenges, CLE successfully highlighted vascular patterns that were distinct to SNUC, including hyperplastic capillaries and the formation of vascular clusters. These features were consistent across CLE imaging and histopathological cross-sections, reinforcing their diagnostic relevance.

#### 4.2.4. Olfactory Neuroblastoma

ONB is a malignant neuroectodermal neoplasm unique to the sinonasal tract, originating from globose basal cells of the olfactory membrane [31]. It accounts for approximately 2% to 3% of all sinonasal neoplasms and is characterized histologically by its composition of small round cells, placing it within the group of small round cell tumors [32]. This group includes a variety of malignancies, many of which share overlapping cytological features, making accurate diagnosis a complex process. ONB’s unique origin from the olfactory membrane often leads to symptoms such as loss of smell, which, combined with the tumor’s typical skull base localization and patient history, can provide valuable clues.

In our study, CLE examination of the ONB case consistently revealed characteristic small, spherical tumor cells, which aligned well with the histopathological findings. However, while the small round cell morphology is a hallmark of ONB, it is not unique to this tumor type. Localization and clinical context play a critical role in narrowing the differential diagnosis.

#### 4.2.5. Sinonasal Malignant Melanoma

SNMM is a rare and aggressive tumor that is often diagnosed at an advanced stage due to its nonspecific symptoms and challenging localization. This malignancy is associated with a high rate of recurrence, exceeding 50%, and a poor prognosis, with a 5-year survival rate of less than 25% [33]. In a preliminary report, our group previously evaluated the applicability and potential advantages of CLE in diagnosing SNMM, highlighting its ability to provide real-time imaging and facilitate surgical decision-making during resection [34].

In the current study, CLE successfully visualized small, spherical tumor cells within the SNMM cases, corresponding well to the histopathological findings. While CLE is not expected to highlight melanin pigments due to their lack of vascularization, we frequently observed a generally darker overall appearance in the CLE images of SNMM tumors compared to other entities, as shown in Figure 9. This raises the question of whether the darker imaging may be indirectly influenced by the presence of melanin pigments within the tumor tissue.

#### 4.2.6. Sinonasal Lymphoma

Hematopoietic neoplasms are among the most common malignancies encountered in the sinonasal tract, with diffuse large B-cell lymphoma being the predominant subtype, as observed in the patient in our study [35] (Denney, J.E., 2024). The diagnosis of sinonasal lymphomas can be challenging due to their variable morphological and immunophenotypic features, which may overlap with those of other malignancies or reactive conditions. These complexities are further compounded by the reliance on small biopsy samples, which can limit the material available for immunostaining.

In our study, CLE demonstrated its potential utility in addressing these diagnostic challenges. The uniform spherical cellular morphology observed in CLE imaging of the lymphoma correlated well with the histopathological findings, and the consistent accumulation of these cells provided a distinguishing feature within our cohort. This real-time imaging capability may help guide biopsies to areas most likely to yield diagnostic material, minimizing the need for repeated sampling due to insufficient or non-representative tissue.

### 4.3. Study Design

This study has several limitations inherent to its design. The limited number of cases reflects the rarity of sinonasal tumors, which constrains the statistical power and generalizability of the findings. Additionally, the pathologists involved in comparing CLE images to histological cross-sections were not blinded to the histological entities. While this may introduce potential bias, the primary aim of this initial study was to characterize the CLE features of each tumor entity as a foundational step toward establishing its utility in differentiating between sinonasal tumors.

Furthermore, the comparison between CLE images and histological findings was purely descriptive, and while we observed notable parallels between the modalities, these correlations must be assessed more rigorously with statistical methods in future studies. Validation of these observations in larger cohorts is essential to evaluate their reproducibility and to determine the predictive power of CLE in reliably diagnosing and distinguishing sinonasal malignancies. These next steps will be critical to advancing the application of CLE as a diagnostic tool in this challenging clinical context.

## 5. Conclusions

This study demonstrates the potential of confocal laser endomicroscopy (CLE) as a diagnostic tool in sinonasal malignancies, providing real-time imaging that correlates well with histopathological findings across various tumor types. By characterizing specific CLE features for entities such as SCC, SC, SNUC, ONB, SNMM, and SL, we highlight the versatility of CLE in addressing the diagnostic challenges posed by these rare and diverse tumors. CLE’s ability to facilitate targeted biopsies and enhance diagnostic precision could reduce the need for repeated sampling and improve workflow efficiency.

However, this study serves as a preliminary step in evaluating CLE’s utility, with limitations including a small sample size, descriptive comparisons, and unblinded analysis. Future research involving larger cohorts, blinded assessments, and robust statistical evaluation is needed to validate our findings and assess the predictive power of CLE in distinguishing sinonasal tumor entities. Despite these limitations, our results underscore CLE’s promise as an adjunctive tool in the diagnostic and therapeutic management of sinonasal malignancies.

## Data Availability

The original contributions presented in this study are included in the article. Further inquiries can be directed to the corresponding author(s).
